# Modeling innovation in the cryptocurrency ecosystem

**DOI:** 10.1038/s41598-022-16924-7

**Published:** 2022-07-28

**Authors:** Giordano De Marzo, Francesco Pandolfelli, Vito D. P. Servedio

**Affiliations:** 1 Centro Ricerche Enrico Fermi, Piazza del Viminale 1, 00184 Rome, Italy; 2 Dipartimento di Fisica Università “Sapienza”, P.le A. Moro, 2, 00185 Rome, Italy; 3 Sapienza School for Advanced Studies, “Sapienza”, P.le A. Moro, 2, 00185 Rome, Italy; 4 Complexity Science Hub Vienna, Josefstaedter Strasse 39, 1080 Vienna, Austria

**Keywords:** Complex networks, Statistical physics

## Abstract

Blockchains are among the most relevant emerging technologies of recent times and, according to many, they will have a central role in shaping the future of our society. Since the introduction of Bitcoin in 2009, the first notorious blockchain system bound to a cryptocurrency, the blockchain ecosystem has experienced a huge growth, driven by innovations both in conceptual and algorithmic terms, and in the creation of a large number of new cryptocoins. New blockchains and their associated cryptocoins, emerge mostly as the result of forking already existing projects. Here, we show that the appearance of new cryptocoins can be well described by a sub-linear power-law (Heaps’ law) of the total crypto-market capitalization. At the same time, we propose a model that well reproduces the evolution of the cryptocurrency ecosystem. Our model suggests that each cryptocurrency triggers, on average, the creation of ca. 1.58 novel cryptocoins, a result confirmed by the analysis of the Bitcoin historical forking tree. Moreover, we deduce that the largest cryptocurrency, nowadays Bitcoin, will comprise around the 50% of the whole crypto-market and that this fraction is going to stabilize in the near future, provided that the present fundamental macro-economic conditions do not change radically.

## Introduction

In the last decade, the blockchain emerged as a breakthrough technology which, according to many, will have a huge impact on our society^1–3^. A blockchain consists of a public decentralized ledger mostly know for being used to record transactions of cryptocurrencies (or cryptos), digital assets that operate as medium of exchange without the need of a central authority^4^. Bitcoin is the first and by no doubt the most famous cryptocurrency. It was introduced by Satoshi Nakamoto (Satoshi Nakamoto is a pseudonym and it is neither clear if it is a single person or a group of people) in 2008^4^, it reached 1000 billion euros of market capitalization (*market cap*, in short) during April 2021 and is currently surfing above 500 billion euros. After the innovative idea of Satoshi Nakamoto not only the financial value of Bitcoin has enormously increased, but many other cryptocurrencies have appeared. Indeed, there are nowadays more than 10,000 cryptos, all based on the original Bitcoin blockchain or on some variants of it, and this number is continuing to increase. Moreover, the blockchain technology is being used for a number of different applications^5^, the most relevant being the Decentralized Finance (or DeFi). It is worth pointing out that despite the potential of this new kind of currency, many financial institutions consider cryptos as a worthless transient and according to some, neither Bitcoin nor the blockchain have succeeded in solving any societal problem^6^. Critics address, for instance, the high price volatility, the huge energy consumption or the long relative time needed for validating transactions and generating liquidity.

**Figure 1:**
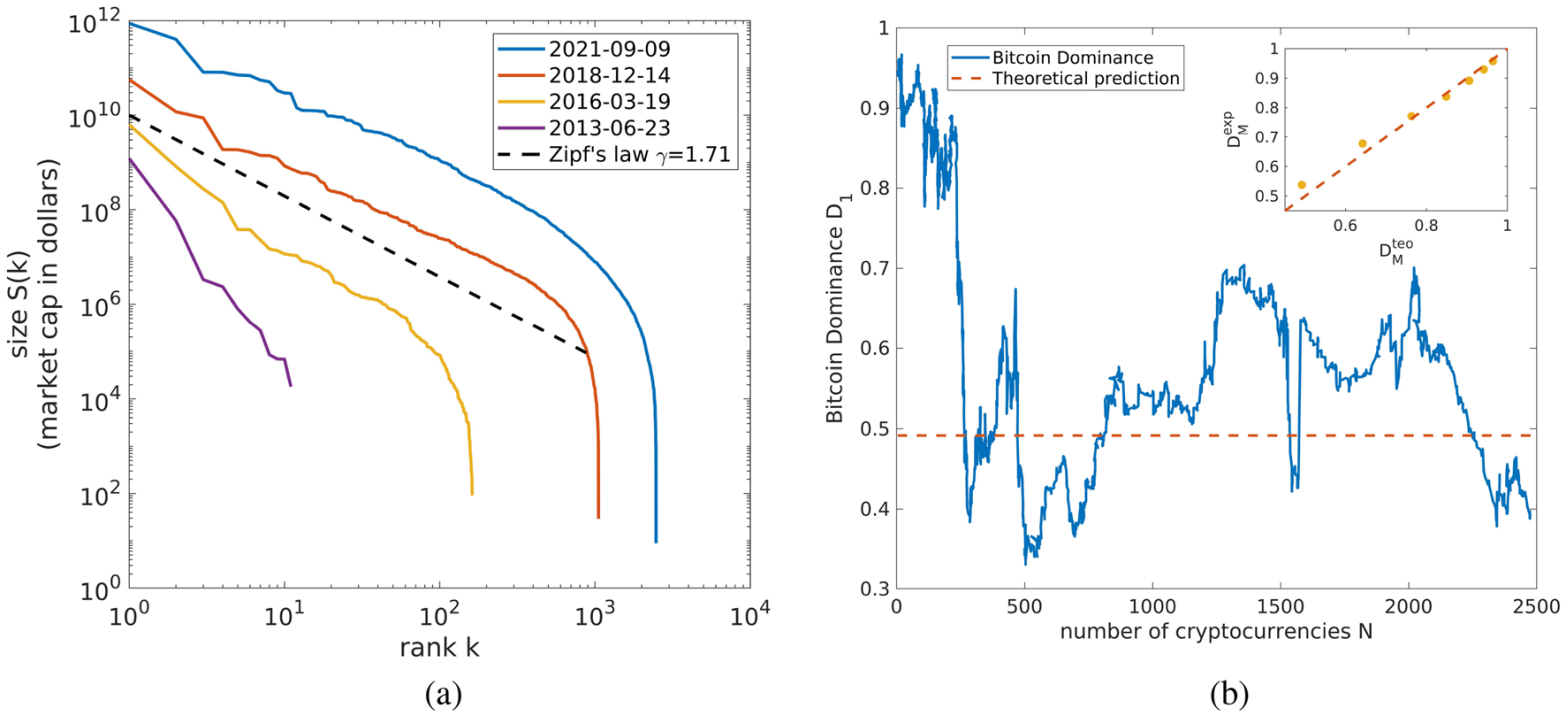
(**a**) Rank-size plots. Evolution of the rank-size plot of the cryptocurrency market over time. The black dashed line represents the expected trend corresponding to the average Zipf’s power law distribution exponent $\gamma \approx 1.71$. (**b**) Dominance of Bitcoin. The Bitcoin dominance is displayed as function of time with the red dashed line showing the asymptotic dominance derived from Zipf’s exponent. Note how the dominance fluctuates around its theoretical estimated value. The inset shows a comparison between the empirical dominance of the first $M=1, 2, 4, 8, 16, 32, 64$ coins and the theoretical values; the red dashed line is the bisector of the quadrant. By considering a larger number of cryptocurrencies, the noise is attenuated and the adherence between theory and empirical data improves. Figures created with Matlab R2021a https://it.mathworks.com/products/matlab.html.

Despite it is still not clear whether cryptos would succeed in revolutionize society, their role is already central in finance and their potential is huge. At present days, the total market cap of cryptocurrencies is approximately 1,500 billions of US dollars and a study carried on in 2019^7^ found out there are about 43 million active crypto traders. It is then not surprising that many studies have tried to analyze cryptocurrencies from a scientific perspective, using tools of complex systems and network science. For instance, several works focused on Bitcoin, with particular attention to its network^8–12^ and its price dynamics or prediction^13–17^, while only few analyses considered the cryptocurrency market in its entirety and tried to model its complex dynamics^18–20^.

Here, we present an analysis that originates from two stylized facts often considered as the footprint of Complexity: Zipf’s law^21^ and Heaps’ law^22^. These laws have been observed in a variety of complex systems such as cities, language, citations of scientific articles, Wikipedia pages and even superclusters of galaxies^23–27^. In the context of cryptocurrencies, Zipf’s law relates the market cap of the *k*th largest coin *S*(*k*) to the capitalization of the first coin *S*(1) as $S(k) = S(1)\cdot k^{-\gamma },$ where $\gamma$ is called Zipf’s exponent and *k* is the rank. Heaps’ law, instead, connects the total market capitalization *n* to the number of different cryptos *N* as $N=n^{\beta }.$ Here $\beta$ is called Heaps’ exponent. Remarkably, the observation of Zipf’s law allows us to assess the existence of two possible regimes the cryptocurrency market can be in, and to determine the asymptotic dominance of the first coin by market cap, currently Bitcoin, which is expected to contribute to approximately $50\%$ of the total crypto market cap in the future. This answers a very relevant question previously considered in literature^19^, i.e. how is Bitcoin dominance evolving and if its decrease is indicative of a future crash, that is still missing a convincing and mathematically grounded answer^20^. Indeed, even if the decrease of dominance has been previously explained as the natural result of an evolutionary model^19^, no conclusions about its asymptotic value have been proposed yet.

While the adherence of cryptos to Zipf’s law has previously been highlighted^19,20^, to the best of our knowledge this is the first time that also Heaps’ law is considered. Heaps’ law is intrinsically connected to innovations^23,28^, which are very likely to occur in the world of cryptocurrencies thanks to their open source, reusable and modular nature^29–31^. For instance, a new blockchain with its cryptocurrency can be created by modifying the (public) code of an already existing blockchain (source code forking) or when a hard fork occurs, therefore making very simple for someone with programming skills to develop their own crypto. The crypto market is thus in constant evolution and very far from being stationary, since new cryptos are introduced every day to solve various aspects as security issues, improving the computational and energetic efficiency of blockchains and offer new decentralized services. In order to mimic such an innovation process, which is one of the key features of the crypto ecosystem, we propose a simple toy model based on Kauffman’s concept of adjacent possible^32^, which, despite its simplicity, is capable of reproducing both Heaps’ and Zipf’s law. We prove this both by means of numerical simulations and analytical computations and we show that the model presents two different regimes depending on the size of the adjacent possible. This allows us to estimate that, on average, each cryptocurrency triggers the birth of less than two, i.e., 1.58 novel criptocurrencies, a result confirmed by the analysis of the historical Bitcoin forking tree.

## Results

### Zipf’s law and the different regimes of the cryptocurrency market

Let us consider a set of *N* cryptocurrencies each characterized by a market cap *S*(*k*) for $k=1\cdots N$. The market cap *S*(*k*) of the *k*th largest cryptocurrency is defined as the number of existing coins times the value of a single coin in a fiat currency (e.g., US dollar or Euro). For instance, at the time of writing this manuscript, a Bitcoin is worth 38, 715 euros and the number of existing Bitcoins is 18, 782, 062, as a result the Bitcoin market cap is approximately 727 billion euros. We say that market cap follows a strict Zipf’s law if it holds

1 $$\begin{aligned} S(k)=\frac{S(1)}{k^{\gamma }} \end{aligned}$$

where $\gamma$ is called Zipf’s exponent and *k* is the rank, i.e., the position of a cryptocurrency in the list of all cryptocurrencies inversely ordered according to their capitalization. The rank-size plot defined in this way is a straight line in double logarithmic scale whenever the system obeys Zipf’s law. In reality, Zipf’s law does not manifest itself as clean as defined by Eq. (1), rather, both fluctuations and discrepancies on the tail of the distribution can be observed, with a power-law behavior holding over three decades in the rank (see Fig. 1a).

The adherence of cryptocurrencies market caps to Zipf’s law and the presence of an inherent power law distribution have already been investigated in Refs.^19,20^. Here, we extend these analyses by considering a wider time period spanning from 2013 to 2021 and follow the procedure described in Ref.^33^ to determine Zipf’s exponent $\gamma$. We measured an average value $\gamma =1.71$ over the period we considered. Figure 1a shows the evolution of the rank-size plot from 2013 to 2021, with the black dashed line denoting the scaling $S(k)\sim k^{-1.71}$ corresponding to the average Zipf’s exponent. As it is possible to see, such a scaling describes the most recent empirical data fairly well.

When considering the cryptocurrency market as a whole, one of the most relevant quantity to be taken into account is the Bitcoin dominance *D*, defined as the fraction of the total market cap held by Bitcoin^19,20,34^. This quantity is often considered a useful indicator to understand the dynamics of the cryptocurrency market and to suggest whether it is better to invest in Bitcoin rather than in other cryptocurrencies^35,36^. At present day, Bitcoin contributes to approximately the $40\%$ of the total market cap of cryptocurrencies, meaning that its current dominance is $D=0.40$. A central question still lacking a definite answer is whether or not Bitcoin will keep its dominant position also in the future^34,37^ or if its dominance will decrease. A diffuse opinion today is that the decrease of dominance observed in the last years could be a sign of decline^38^, while some others believe that Bitcoin dominant role is not in danger at all^39^. Trying to answer this key question, in^19^ it was found out that Bitcoin dominance decreased linearly in time from 2013 to 2017 with the prediction that the dominance would have reached 50% by year 2025. While this value has already been reached some years ago, it is not clear whether this linear decrease would keep going till zero or if Bitcoin will asymptotically tend to a non null fraction of the total market cap.

In our framework the dominance of the largest cryptocoin, currently Bitcoin, is given by

$$\begin{aligned} D_1=\frac{S(1)}{n}, \end{aligned}$$

where, as above, *S*(1) is the largest market cap and *n* the total market cap. We can obtain *n* by summing over all the capitalization of the *N* cryptocurrencies, that is

$$\begin{aligned} n=\sum _{k=1}^N S(k)=S(1)\sum _{k=1}^N \frac{1}{k^{\gamma }}, \end{aligned}$$

where we used Zipf’s law Eq. (1) to recast *S*(*k*). The dominance as function of *N* is thus

2 $$\begin{aligned} D_1(N, \gamma )=\frac{1}{\sum _{k=1}^N \frac{1}{k^{\gamma }}} \end{aligned}$$

and taking the limit $N\rightarrow \infty$ we get its asymptotic value

3 $$\begin{aligned} D_1=\lim _{N\rightarrow \infty }D_1(N, \gamma )=\frac{1}{\zeta (\gamma )}, \end{aligned}$$

where $\zeta (\gamma )$ is the Riemann zeta function evaluated in $\gamma$. Remarkably, Eq. (3) shows that depending on Zipf’s exponent $\gamma$ there are two different possible regimes as both *n* and *N* grow

- If $\gamma \le 1$ the zeta function is diverging and this implies that the asymptotic dominance is null
  $$\begin{aligned} \lim _{N\rightarrow \infty }D_1(N, \gamma )=0. \end{aligned}$$
  In other words, in the limit of an infinite system the largest cryptocurrency holds only an infinitesimal fraction of the total market cap.
- If $\gamma >1$ the zeta function is a finite number meaning that the asymptotic dominance is strictly larger than zero
  $$\begin{aligned} \lim _{N\rightarrow \infty }D_1(N, \gamma )>0. \end{aligned}$$
  In other words, in the limit of an infinite system the largest cryptocurrency holds a non null fraction of the total market cap.

In the system we are considering $\gamma \approx 1.71>1$, so that we are in the second regime and we expect Bitcoin to hold a finite fraction of the total capitalization even in the asymptotic limit. More precisely, using Eq. (3), we get

$$\begin{aligned} D_1\approx 0.5 \end{aligned}$$

Figure 1b shows a comparison between the observed dominance of Bitcoin as function of *N* and this theoretical prediction. We note that we have already reached the asymptotic value and Bitcoin dominance is oscillating around it. It is important to remark that the result we derived applies to the top cryptocurrency and not specifically to Bitcoin. Indeed, there is a finite probability of turnover^19^, meaning that in the future Bitcoin could lose its position in favor of another coin, even if it has been shown that top cryptos tend to be more stable and less prone to turnovers^19^. Also, we note that the estimate of the asymptotic dominance depends on the the power law exponent, which has been evolving in the time period considered (see Fig. 6 in the Methods section) and appears to be stable since 2018. As a consequence, this estimate can be considered reliable only provided that the present fundamental macro-economic conditions and the exponent do not change radically. In order to asses the stability of the asymptotic dominance, we computed it only considering data up to the end of 2018. In this way we obtained $D_1\approx 0.55$, a value $10\%$ higher than the previous estimate. This should give an idea of how much the asymptotic dominance can vary in approximately 2 years as the result of the fluctuations of the Zipf’s exponent.

We can compute the dominance of the first *M* cryptocurrencies as well, as

$$\begin{aligned} D_M(N, \gamma )=\frac{\sum _{k=1}^M S(k)}{\sum _{k=1}^N S(k)} \end{aligned}$$

and for $N\rightarrow \infty$ we get

4 $$\begin{aligned} D_M=\lim _{N\rightarrow \infty }D_M(N, \gamma )=\frac{H_M^{(\gamma )}}{\zeta (\gamma )}, \end{aligned}$$

where $H_M^{(\gamma )}$ is the generalized harmonic number of order $\gamma$ of *M*. As in the case of $D_1$, also here all the $D_M$ are null for $\gamma \le 1$, while they are larger than zero if $\gamma >1$. The inset of Fig. 1b shows a comparison between the empirical asymptotic values of $D_M$ for $M=1, 2, 4, 8, 16, 32, 64$ and Eq. (4). We observe that by increasing *M*, the noise is averaged out and so the adherence to theory gets better and better. The key message of this analysis is that the decrease of Bitcoin dominance observed since 2009 can be fully explained by the inherent power law distribution and the increase of the total market cap. The decrease of *D* is thus not surprising and could not be related to a sign of Bitcoin decline.

**Figure 2:**
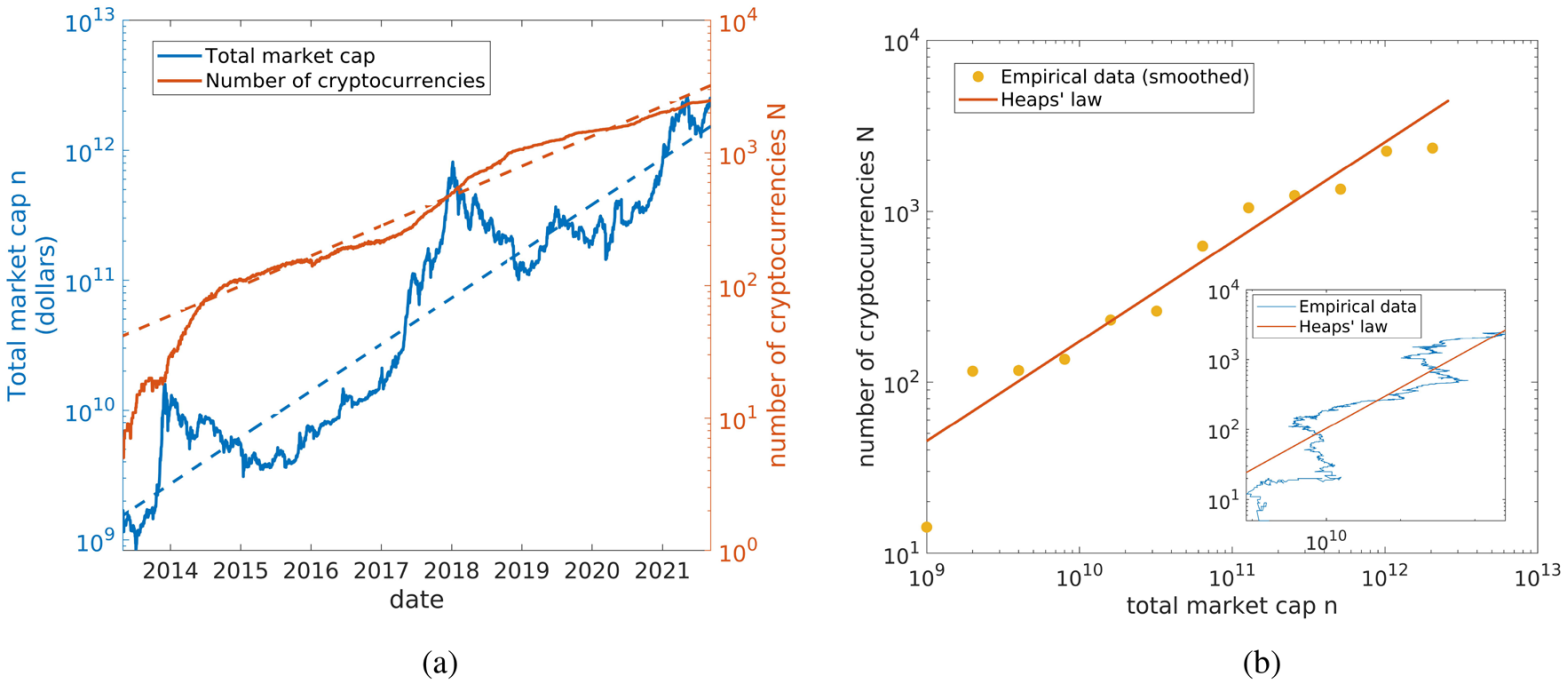
(**a**) Evolution of the number of different coins *N* and market cap *n* over time. Growth of the number of coins *N* (red line) and of the total market cap *n* (blue line) as function of time. Both quantities have increased at an exponential rate in time since 2013. Dashed lines are guides for the eyes. (**b**) Heaps’ law. Parametric plot of *N* versus *n*; the yellow points have been obtained performing an average with exponentially increasing window. The number of coins grows as a sublinear power-law of the total market cap, indicating the presence of Heaps’ law in the market cap. Red line shows the theoretical Heaps’ law computed from Zipf’s exponent. We also reported in the inset the raw empirical data without averaging. Figures created with Matlab R2021a https://it.mathworks.com/products/matlab.html.

### Heaps’ law: innovation in the cryptocurrency ecosystem

Heaps’ law is another scaling law that is often found in complex systems. Initially observed in linguistics, this law states that the number of distinct words *N* in a text grows as function of the total number of words *n* as

5 $$\begin{aligned} N(n)=n^{\beta }. \end{aligned}$$

Here $\beta$ is called Heaps’ exponent which, when also Zipf’s law is observed, is related to Zipf’s exponent by the relation

6 $$\begin{aligned} \beta = {\left\{ \begin{array}{ll} \frac{1}{\gamma }\ \quad {\text {for}}\ \gamma >1\\ 1\ \qquad {\text {for}}\ \gamma <1. \end{array}\right. } \end{aligned}$$

It is worth pointing out that deviations from this relation arise for finite *N* and in presence of an upper cutoff in the power law scaling^24,25^. Heaps’ law is intrinsically connected to innovation processes and novelties occurring in the system^23^. Indeed, the rate of innovation $\rho$, that is the rate at which new elements enter the system, is obtained deriving *N* with respect to *n*, which can be seen as a playing the role of a temporal variable

$$\begin{aligned} \rho =\frac{dN}{dn}=n^{\beta -1}. \end{aligned}$$

This implies that when a sublinear Heaps’ law is observed the innovation rate decreases as the system evolves.

Moving from words in a text to cryptocurrencies, we have that *N* coincides with the number of different cryptos, while *n* is equivalent to the total market cap. Note that this last quantity can in principle also decrease in time, but, neglecting fluctuations, it has been increasing on average since the birth of the first cryptocurrency. In order to determine whether or not the growth of this system can be described by Heaps’ law as well, we considered the the cryptocurrencies ecosystem in the period 2013–2021. Figure 2a shows the evolution of *n*(*t*) and *N*(*t*) over this period, while the parametric plot of *N*(*t*) vs *n*(*t*) is shown in Fig. 2b. As it is possible to see, the cryptocurrency ecosystem is well described by Heaps’ law and, as a consequence, the number of cryptocurrencies *N* and the total market cap *n* do not evolve independently. In fact, as the total market cap grows, the number of cryptocurrencies has to grow in response according to the law $N(t)=n(t)^{\beta }$. Note that while the rank-size plot and Zipf’s law provide a snapshot of the system at a given moment, Heaps’ law describes its dynamics as it evolves. The solid line in the main figure and in the inset represents Heaps’ scaling with exponent $\beta$ given by the reciprocal of Zipf’s exponent that we computed previously

7 $$\begin{aligned} \beta = \frac{1}{\gamma }=0.58. \end{aligned}$$

Since Heaps’ exponent is smaller than one, the cryptocurrency ecosystem is characterized by an innovation rate which decreases as the total market cap increases. Finally, it should be noticed that in order for Eq. (6) to hold exactly, the lower cutoff of the probability distribution, that is the smallest market cap, must be constant on average^24^. This is approximately true for the system under consideration and we also checked that by introducing a cutoff by hand, that is by considering all coins above a fixed threshold $x_{min}$, results do not change. In particular we considered $x_{min}=10^2$, $10^3$ and $10^4$ and we obtained only negligible differences with respect to Fig. 2b.

**Figure 3:**
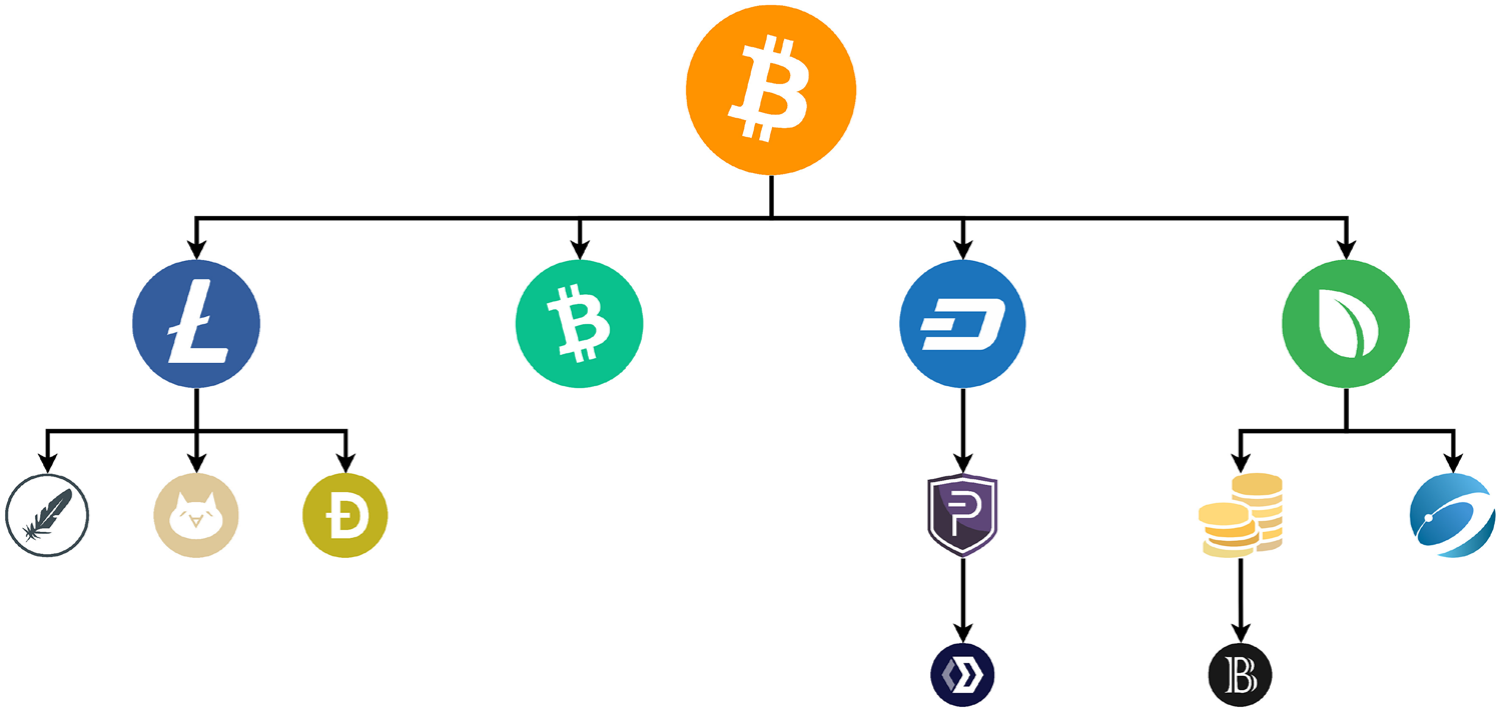
Forks of Bitcoin. Schematic representation of Bitcoin forking tree. Each time a new cryptocurrency is created, its code (software fork) or its blockchain (hard fork) can be used as a starting point for the creation of new coins and blockchains. For instance Litecoin has been created by minor modifications of Bitcoin code and in its turn Litecoin source code has been used to develop many other coins such as the well known Dogecoin. The creation of new cryptocurrencies can be described in terms of an adjacent possible process. Figure created with www.diagrams.net.

### Modeling innovation in the cryptocurrency ecosystem

As we mentioned in the introduction, innovation is one of the driving forces of the cryptocurrency ecosystem mainly due to the open source nature of blockchains. Since all the source code related to a cryptocurrency is typically publicly available, a new blockchain with its own crypto can be created by modifying or combining such source codes. For instance Litecoin was created in 2011 starting from Bitcoin source code by modifying the block generation time, the maximum number of coins and the hashing algorithm, but most of its structure is equal to that of Bitcoin. Analogously, the source code of Litecoin was then used as a starting point for the making of Dogecoin, Auroracoin and many other cryptocurrencies. Such processes are usually called software forks, but a new cryptocurrency can be created also by a so called hard fork or blockchain fork. A hard fork occurs whenever some nodes participating to the blockchain decide not to uniform to an update of the rules governing the blockchain or to adopt new rules. This makes the blockchain split and leads to the birth of a new cryptocurrency. Examples are Ethereum Classic, which resulted from an hard fork of Ethereum blockchain in 2016, or Bitcoin Cash, originated from Bitcoin blockchain in 2017. Finally, new cryptocurrencies can be created by copying parts of the source code of already existing coins, a practice that has been particularly common in recent years^30,31^. In the following, we shall make no distinction between the processes we just described, since their result is substantially the same, i.e., the creation of a new cryptocurrency by exploiting the resources available from previous projects. Once a new cryptocurrency comes to life, it can undergo further forks and become, on its turn, the starting point for the creation other cryptos, as schematized in Fig. 3.

This innovation process we just described, very much resembles to the concept of adjacent possible introduced by Kaufman^32^ to describe biological evolution. In Kaufman’s theory, the adjacent possible is the set of all those things (real or abstract), in our case cryptocurrencies, which have not yet been created, but that are close to become reality. As soon as something moves from the adjacent possible to real life, other objects get closer to be realized and thus enter the adjacent possible that consequently expands. As a consequence, innovation can be seen as the process of exploring the adjacent possible. The cryptocurrency ecosystem grows in the very same way, since, as we described above, when a technological improvement is achieved and a novel crypto is created, other developers can use its source code as a starting point for new cryptocurrencies.

**Figure 4:**
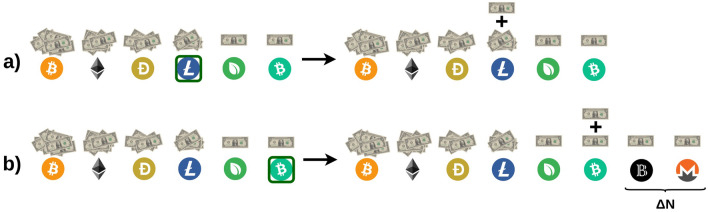
Schematic representation of the model. At each time step a unit of money is invested in a cryptocurrency selected with a probability proportional to its market cap, which is then increased by a unit. If such a cryptocurrency previously received other investments we move to the next time step (case a), while if it is the first time the cryptocurrency is selected we also introduce in the system $\Delta N$ novel coins with unitary capitalization (case b). This last process mimics the adjacent possible mechanism behind the creation of new cryptocurrencies. Figure created with www.diagrams.net.

Our model is thus based on one side on the concept of adjacent possible for describing how innovation takes place, and on the other on the preferential attachment mechanism to schematize how existing cryptocurrencies grow^20^. The steps of the model are the following

- initially there is only one cryptocurrency with unitary market cap;
- at each time step a cryptocurrency is randomly selected with a probability proportional to its market cap and a unit of money is added to it. This process schematize a person investing in a given cryptocurrency;
- when a cryptocurrency receives its first investment the creation of $\Delta N$ novel cryptocurrencies with a unitary capitalization is triggered. This mimics the adjacent possible in the innovation process of the cryptocurrency ecosystem.

Figure 4 is a schematic representation of this model, one can see the two different update events: the first row (a) shows an investment in a cryptocurrency that previously received other investments, while the second row (b) corresponds to an investment in a novel cryptocurrency followed by the creation of $\Delta N$ new cryptos. Despite its simplicity, the model here presented shows both Zipf’s and Heaps’ law and the corresponding exponents depend on the value of $\Delta N$, more precisely it holds

8 $$\begin{aligned} \gamma _{mod}=\frac{1}{\Delta N-1} \end{aligned}$$

and, consistently with Eq. (6) Heaps’ exponent satisfies

9 $$\begin{aligned} \beta _{mod}= {\left\{ \begin{array}{ll} \Delta N-1 \ {\text {if}}\ \Delta N<2\\ \quad \quad 1\ \quad {\text {if}}\ \Delta N>2 \end{array}\right. } \end{aligned}$$

**Figure 5:**
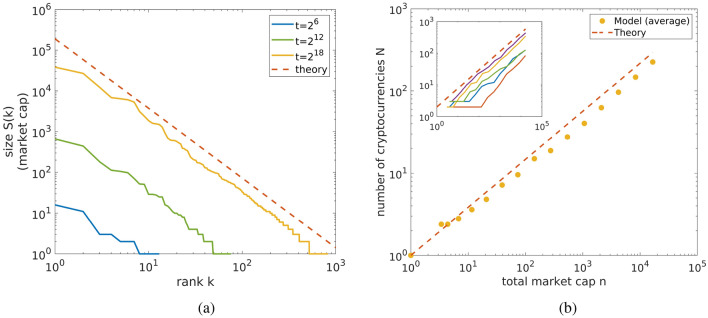
(**a**) Rank-size plots. Rank-size plots obtained from our model with $\Delta N=1.58$. The model produces a Zipf’s law with exponent given by $1/(\Delta N-1)$; the red dashed line is the theoretical prediction. (**b**) Heaps’ law. Growth of the number of coins in the model as function of the market cap. Yellow points have been obtained averaging over 5 different realizations (shown in the inset), while the red dashed lines is the theoretical Heaps’ law with exponent $\Delta N-1$. Figures created with Matlab R2021a https://it.mathworks.com/products/matlab.html.

The interested reader can find a derivation of these relations and a proof that the model presented here is asymptotically equivalent to the model proposed by Ref.^23^ in the Methods section. Figure 5 shows a comparison between the theoretically predicted Heaps’ and Zipf’s scalings and those obtained by numerical simulations of the model. Equations (8) and (9) are in good agreement with the simulations. It is worth remarking that the model we introduced has some similarities with the evolutionary model proposed in Ref.^19^. In both cases the growth of existing cryptocurrencies is driven by a rich-get-richer mechanism, but the two models differs on how new coins enter the system. Indeed, in the evolutionary model there is a fixed mutation parameter governing the creation of new currencies and thus the birth of a cryptocoin is a completely random event. This is a major difference with respect to our model, where the creation of a novel crypto is triggered by another coin getting its first investment, mimicking an adjacent possible mechanism. Moreover, the evolutionary model always gives rise to a Zipf’s law with exponent 1.5 independently of the mutation parameter, while in our model the size of the adjacent possible determines Zipf’s exponent and the two different possible regimes accordingly. In essence, both models are conceptually similar, with the adjacent possible framework that was originally proposed to explain biological evolution. We think these similarities strongly indicate that the cryptocurency market can be successfully described as an evolving ecosystem, even if the specific mechanisms can vary from model to model.

Equations (8) and (9) show that in the model we just described, $\Delta N$ determines in which of the two regimes the system is. Since we know that the cryptocurrency market is characterized by a Zipf exponent larger than one, the model thus predicts an adjacent possible $\Delta N$ less than two. In other words, according to our model, the creation of a new cryptocurrency should trigger, on average, the birth of less than two novel cryptocurrencies. In order to measure if this is actually the case in reality, we considered the forking tree of Bitcoin created by www.mapofcoins.com and available on GitHub^40^. This dataset, which covers the period 2009–2015, contains 661 different cryptocurrencies, each linked to the cryptocurrency from which it has been forked. Note that many of these cryptocurrencies are not listed on coinmarketcap.com and this means that they never succeed in getting relevant investments. In order to measure the size of the adjacent possible of this forking tree, in analogy with our model, we selected among the 661 available cryptos all those listed on coinmarketcap.com, for a total of 241 different cryptocurrencies, and then we defined the adjacent possible as the average number of forks produced by such coins. Note that in computing the average adjacent possible dimension we include all coins, and only those coins that have been traded at least once contribute to it.

In other words we compute the average adjacent possible as

$$\begin{aligned} \Delta N_{\exp }=\frac{1}{N_0}\sum _{i=1}^{N_0}\Delta N_i, \end{aligned}$$

where $N_0=241$ is the number of cryptocurrencies that appears in the forking tree and have also been traded, while $\Delta N_i$ is the number of forks produced by the *i*-th coin. This eventually results in

10 $$\begin{aligned} \Delta N_{\exp }=1.59. \end{aligned}$$

This number is remarkably close to what one would expect from real data, since Eqs. (7) and (9) give $\Delta N_{\mathrm {teo}}=1.58$. It is important to remark that the forks dataset we used is quite limited, since many of the cryptocoins that nowadays rank first have been created after its release. Moreover, the capitalization dataset we used starts in 2013, so the overlap between the two datasets is only partial, and the number of coins it contains is much larger. However, even if we limit Zipf’s and Heaps’ analysis to the coins contained in the forking tree of Bitcoin and we only consider data up to 2015, we still obtain a Zipf’s exponent larger than one (2.44) and so also in this case the model successfully predict the correct regime, even if in this case $\Delta N_{\mathrm {teo}}=1.44$ which is $10\%$ smaller than the empirical value. These results suggest that the simple model we introduced, captures the mechanism behind the innovation process in the cryptocurrency ecosystem.

## Discussion

Since the birth of Bitcoin in 2009, the cryptocurrency market has experienced a huge growth both in terms of the total market cap and in the number of different cryptocurrencies. One of the driving forces of such an expansion has been innovation, since all the source code of cryptos and blockchains is typically public and can thus be reused and composed to create novel cryptocoins. During this process the cryptocurrency market has been characterized by some stable statistical regularities, one of them being Zipf’s law. Starting from this, we showed that depending on the value of Zipf’s exponent there are two possible regimes in which the cryptocurrency market can be. If Zipf’s exponent is smaller than one, than the dominance (or market share) of the largest cryptocoin asymptotically goes to zero, while if Zipf’s exponent is larger than one, as observed in real data, the dominance is asymptotically larger than zero. This analysis also allowed us to compute the expected asymptotic dominance of Bitcoin, which we believe will keep oscillating around the value 0.5. Furthermore, we showed that the growth of the cryptocurrency ecosystem can be described in terms of Heaps’ law, a statistical law often encountered in open expanding systems characterized by novelties and innovation. In the case of cryptos, this law implies that the number of distinct cryptocurrencies grows as a sub-linear power of the total market cap, thus showing that these two quantities are intrinsically related. Finally, we proposed a model to describe the innovation process in the cryptocurrency ecosystem based on preferential attachment and on the concept of adjacent possible. The model reproduces both Heaps’ and Zipf’s law and the presence of two different regimes depending on how many cryptocoins are triggered by the creation of a novel cryptocurrency, that is depending on the size of the adjacent possible. In particular, the regime in which the dominance of the largest crypto is larger than zero corresponds to an adjacent possible smaller than two. Being more precise, the model predicts this number to be equal to 1.58 and analyzing the forking tree of Bitcoin we found out that in this case the average size of the adjacent possible is 1.59. Despite this striking accordance, we stress that our model still has some limits. Indeed, while we assumed each crypto to trigger the same number of novel cryptocurrencies, in the forking tree of Bitcoin there are some coins, such as Litecoin and Bitcoin itself, which are responsible for a large fraction of the total number of coins. It would be thus interesting to consider a probability distribution for the size of the adjacent possible in order to mimic also this aspect. Moreover, the data we used to measure the adjacent possible are limited to the forking tree of Bitcoin and do not cover recent years, analyzing also other forking trees and/or more recent data would by no doubt give additional insight on the process of innovation in the cryptocurrency ecosystem.

## Methods

### Fitting procedure

**Figure 6:**
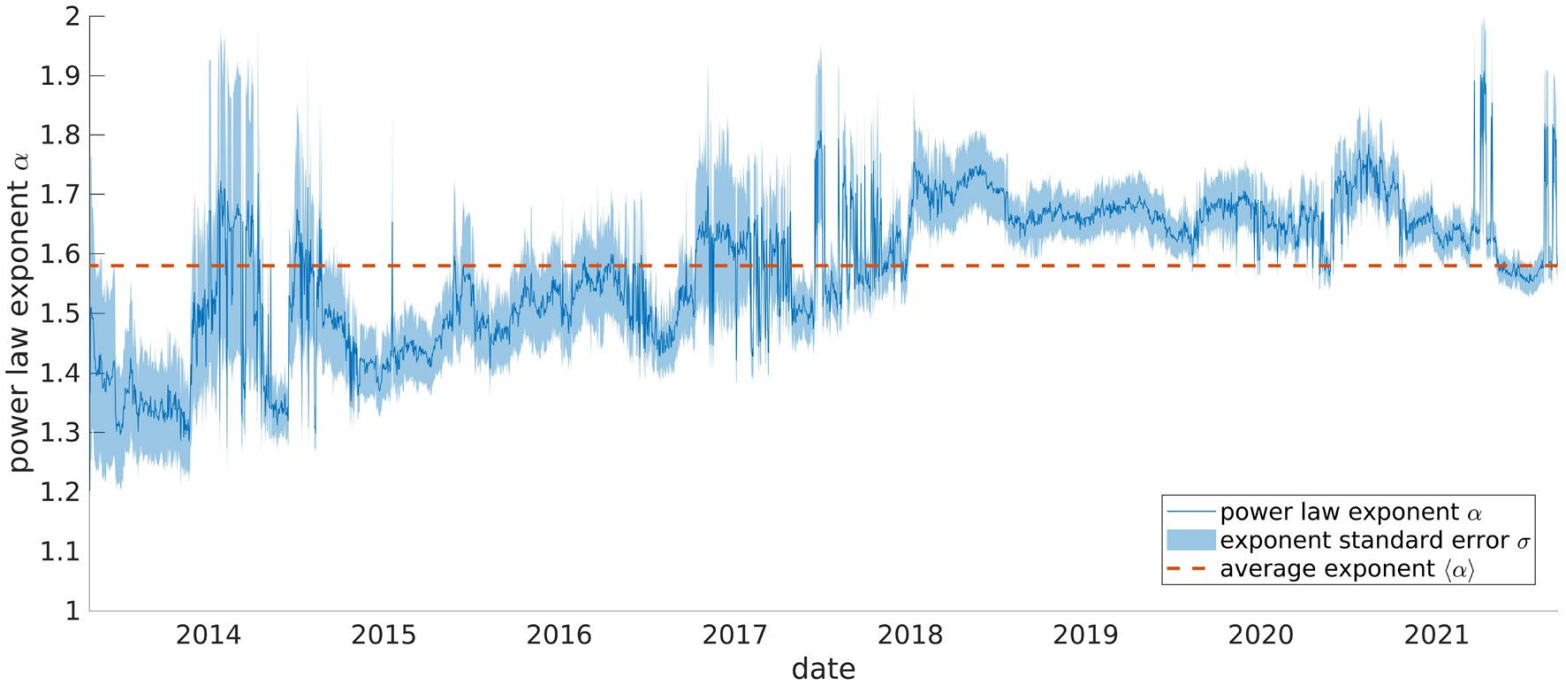
Power law exponent. Exponent of the power law distribution of market caps as function of time (blue line) and of its standard error (shaded area), both computed with a maximum likelihood approach. As the time elapses the error decreases since the number of coins over which the fit is performed increases. The red dashed line shows the average exponent obtained as an average weighted by the standard error. Figure created with Matlab R2021a https://it.mathworks.com/products/matlab.html.

In order to compute the average Zipf’s exponent of the cryptocurrency market we started by computing the exponent of the underlying probability distribution $P(S)\sim S^{-\alpha }$ for all the days in the period 2013/04/28–2021/09/09. This has been done using the python powerlaw package^41^ which implements the technique described in^33^. In few words this method exploit the maximum-likelihood fitting technique and the Kolmogorov-Smirnov statistic to asses both the exponent of the power law probability distribution $\alpha$ with its standard error $\sigma$ and the lower cutoff where the power-law behavior ceases to hold. Note that in principle one should take into account also the possible presence of an upper cutoff in the power law distribution, though the system under consideration does not present such a cutoff. This can be seen by noticing that the rank-size plots never shows a negative curvature at low ranks^27^. In this way for each day *t* we obtained the exponent $\alpha _t$ and its standard error $\sigma _t$. During the first years of our dataset only few cryptocurrencies were present and so the estimate of the power law exponent is less reliable and is affected by a high level of uncertainty, while the $\alpha _t$ computed over more recent data are characterized by a much lower error. This is can be clearly seen in Fig. 6, where we plotted $\alpha _t$ with its uncertainty. As a consequence, we computed the mean power law exponent $\langle {\alpha }\rangle$ as an average weighted with the standard error

$$\begin{aligned} \langle {\alpha }\rangle =\frac{\sum _t \frac{\alpha _t}{\sigma _t}}{\sum _t \frac{1}{\sigma _t}} \end{aligned}$$

and we obtained

$$\begin{aligned} \langle {\alpha }\rangle =1.58. \end{aligned}$$

Starting from the power law exponent, Zipf’s exponent $\gamma$ can be easily computed by the relation $\gamma =1/(\alpha -1)$ (see for instance^24^ and references therein) and so we ended up with the value

$$\begin{aligned} \gamma = 1.71. \end{aligned}$$

### Mathematical description of the model

Here we provide a mathematical description of the adjacent possible based model we introduced above. We recall that the model is defined as follows:

- at $t=0$ the system contains a single cryptocurrency with unitary market cap;
- at each time step a cryptocurrency is selected with probability proportional to its market cap, which is then increased by a unit;
- when a crypto receives its first investment $\Delta N$ new cryptocurrencies with unitary market cap are added to the system.

We begin computing how the number of different cryptocurrencies evolves in time *t*. We denote by $N_0(t)$ the number of cryptos which have received at least an investment, in these terms the total number of cryptocurrencies in the system is

$$\begin{aligned} N(t)=1+N_0(t)\cdot \Delta N\approx N_0(t)\cdot \Delta N. \end{aligned}$$

Note that the $+1$ derives from the fact that initially there is only one cryptocurrency in the system; we neglect this term since we are interested in the large *N*(*t*) behavior. The probability of increasing $N_0(t)$ of a unit is then

$$\begin{aligned} P(N_0(t)\rightarrow N_0(t+1)=N_0(t)+1) = \frac{N(t)-N_0(t)}{n(t)}, \end{aligned}$$

where the numerator gives the number of cryptocurrencies which never received an investment, while the denominator is the total market cap. Noting that

$$\begin{aligned} n(t)=t+N(t) \end{aligned}$$

and using the expression for $N_0(t)$ we thus obtain

$$\begin{aligned} P(N_0(t)\rightarrow N_0(t+1)=N_0(t)+1) = \frac{(\Delta N-1)N_0(t)}{t+\Delta N\cdot N_0(t)}. \end{aligned}$$

As a consequence the differential equation governing the growth of $N_0(t)$ is

11 $$\begin{aligned} \frac{d N_0(t)}{dt}=\frac{(\Delta N-1)N_0(t)}{t+\Delta N\cdot N_0(t)}. \end{aligned}$$

We have two different possibilities

- if we assume $t\gg N_0(t)$ we can approximate Eq. (11) as
  $$\begin{aligned} \frac{d N_0(t)}{dt}=\frac{(\Delta N-1)N_0(t)}{t} \end{aligned}$$
  whose solution is
  $$\begin{aligned} N_0(t)=t^{\Delta N-1} \ \rightarrow \ N(t)=\Delta N t^{\Delta N-1}. \end{aligned}$$
  This solution is compatible with the assumption $t\gg N_0(t)$ provided that $\Delta N<2$.
- conversely if $t\sim N_0(t)$ so that $N_0(t)=ct$ we have from Eq. (11)
  $$\begin{aligned} c=\frac{(\Delta N-1)c}{1+c\Delta N} \ \rightarrow \ c=\frac{\Delta N-2}{\Delta N} \end{aligned}$$
  which gives
  $$\begin{aligned} N(t)=\Delta N ct=(\Delta N-2)t \end{aligned}$$
  This expression is meaningful provided that $\Delta N>2$.

Concluding, as stated in Eq. (9), we have two distinct regimes

12 $$\begin{aligned} N(t)= {\left\{ \begin{array}{ll} \Delta N t^{\Delta N-1} \ {\text {for}}\ \Delta N<2\\ (\Delta N-2)t \ {\text {for}}\ \Delta N>2, \end{array}\right. } \end{aligned}$$

this proving that the model shows Heaps’ law.

We can now turn to Zipf’s law, first we write the expression governing the growth of a given crypto, let us say the *k*th. The evolution of its capitalization *S*(*k*, *t*) as function of *t* is given by

$$\begin{aligned} \frac{dS(k, t)}{dt} = \frac{S(k,t)}{n}=\frac{S(k,t)}{t+N(t)} \end{aligned}$$

whose solution, using Eq. (12), is

$$\begin{aligned} S(k, t) = {\left\{ \begin{array}{ll} \frac{t}{t_k}\ {\text {for}} \ \Delta N<2\\ ({\frac{t}{t_k}})^\frac{1}{\Delta N-1}\ {\text {for} }\ \Delta N>2. \end{array}\right. } \end{aligned}$$

Here, we denoted by $t_k$ the time at which the *k*th coin entered the market. Considering the first case $\Delta N<2$ we can then express the cumulative probability of *S*(*k*) starting from that of $t_k$ as

$$\begin{aligned} P(S(k)<S)=P(t_k>t/S)=1-P(t_k<t/S)\approx \frac{N(t/S)}{N(t)}\sim \frac{1}{S^{\Delta N -1}}. \end{aligned}$$

Analogously for $\Delta N>2$ we obtain

$$\begin{aligned} P(S(k)<S)=1-P(t_k<t/S^{\Delta N-1})\sim \frac{1}{S^{\Delta N-1}}. \end{aligned}$$

Since $P(S(k)<S)\sim S^{-\Delta N-1}$ the rank-size relation of *S*(*k*) is of the form

$$\begin{aligned} S(k)\sim k^{\frac{1}{\Delta N-1}}. \end{aligned}$$

This confirms Eq. (8).

Finally, we note that the model we studied is equivalent to a Polya urn: different cryptocurrencies correspond to balls of different colors, while the total market cap of a coin is equivalent to the number of balls of its color contained in the urn. At each time step a ball is extracted from the urn and then it is put back together with a copy of it. Moreover, if the ball is of a never seen color, also $\Delta N$ balls of novel colors are inserted in the urn. Such a model has been studied in^23^, but while they focused on the sequence of extracted balls, here we consider the content of the urn. Denoting by $n'$ and $N'$ the quantities obtained in^23^, they can be related to those we computed, *n* and *N*, by the following relations

$$\begin{aligned} {\left\{ \begin{array}{ll} N'(t) = \frac{N(t)}{\Delta N}\\ n'(t) = n(t)-N(t). \end{array}\right. } \end{aligned}$$

Since *N*(*t*) scales at most as *n*(*t*), these expressions imply that for large *t* it holds $N(t)\sim N'(t)$ and $n'(t)\sim n(t)$ and so the two models show the same Heaps’ law. For what concerns Zipf’s law, we have $S'(k)=S(k)-1$ and so $S'(k)\approx S(k)$ provided that *k* is sufficiently large. As a consequence, the two models are characterized by the same statistical properties.

### Data

All the data we used in the paper can be accessed on the web.

- Historical market cap data have been downloaded from https://coinmarketcap.com/. The dataset we downloaded cover the period 2013-04-28 to 2021-09-09 and contains a total of 4588 cryptocurrencies (at the time of download). For each coin we only retained market capitalization data, but also other information are available. In particular we downloaded the market capitalization of each coin for each day in the period mentioned above, for a total of 3057 days.
- Forks data can be visualized at www.mapofcoins.com and we downloaded the raw data from https://github.com/Ada-Alternative/Mapofcoins/blob/master/coins-btc.json. The dataset covers the period 2009–2015 and contains 661 different cryptocurrencies, all originated, directly or indirectly, from Bitcoin. For each coin different information are available, such as the announce and genesis dates, the PoW or PoS algorithm used or the block time. Moreover, each crypto is also linked to the cryptocurrency it has been forked from and this allows to reconstruct the forking tree of Bitcoin up to 2015.
